# Resistance to Molecularly Targeted Therapies in Melanoma

**DOI:** 10.3390/cancers13051115

**Published:** 2021-03-05

**Authors:** Meet Patel, Adam Eckburg, Shahina Gantiwala, Zachary Hart, Joshua Dein, Katie Lam, Neelu Puri

**Affiliations:** Department of Biomedical Sciences, University of Illinois College of Medicine at Rockford, Rockford, IL 61107, USA; mpate307@uic.edu (M.P.); adam-eckburg@northwestern.edu (A.E.); sganti3@uic.edu (S.G.); zhart4@uic.edu (Z.H.); jdein2@uic.edu (J.D.); klam23@uic.edu (K.L.)

**Keywords:** melanoma, molecularly targeted therapies, TKIs, resistance, biomarkers

## Abstract

**Simple Summary:**

Metastatic melanoma has poor survival rates in comparison to other types of skin cancer. Traditional therapies are not very effective in treating advanced melanoma. For this reason, several molecular targeted therapies such as tyrosine kinase inhibitors are currently being used to treat metastatic melanoma; however, patients quickly develop resistance to these therapies. In this review, we comprehensively discuss various biomarkers and their potential mechanisms of acquired resistance to tyrosine kinase inhibitors.

**Abstract:**

Malignant melanoma is the most aggressive type of skin cancer with invasive growth patterns. In 2021, 106,110 patients are projected to be diagnosed with melanoma, out of which 7180 are expected to die. Traditional methods like surgery, radiation therapy, and chemotherapy are not effective in the treatment of metastatic and advanced melanoma. Recent approaches to treat melanoma have focused on biomarkers that play significant roles in cell growth, proliferation, migration, and survival. Several FDA-approved molecular targeted therapies such as tyrosine kinase inhibitors (TKIs) have been developed against genetic biomarkers whose overexpression is implicated in tumorigenesis. The use of targeted therapies as an alternative or supplement to immunotherapy has revolutionized the management of metastatic melanoma. Although this treatment strategy is more efficacious and less toxic in comparison to traditional therapies, targeted therapies are less effective after prolonged treatment due to acquired resistance caused by mutations and activation of alternative mechanisms in melanoma tumors. Recent studies focus on understanding the mechanisms of acquired resistance to these current therapies. Further research is needed for the development of better approaches to improve prognosis in melanoma patients. In this article, various melanoma biomarkers including BRAF, MEK, RAS, c-KIT, VEGFR, c-MET and PI3K are described, and their potential mechanisms for drug resistance are discussed.

## 1. Introduction

Presently, skin cancer is the most common type of cancer in humans, while melanoma ranks as the most lethal form of skin cancer. Despite the fact that melanoma only represents about 1% of total skin cancers, it leads to the majority of skin cancer-related deaths [1,2]. Melanoma is the result of melanocyte damage from overexposure to UV light or radiation. The resulting DNA damage leads to cancer causing mutations, uncontrolled cell growth, and tumorigenesis. When diagnosed in the early stages, it is possible to treat melanoma through surgery, leading to longer survival rates. However, once the melanoma spreads to distant organs, treatment options are limited [3]. Chemotherapy provides little therapeutic benefit to patients with malignant melanoma, demonstrated by a limited response rate of 10–15% to the standard chemotherapeutic dacarbazine [4]. According to the American Cancer Society, the 5-year survival rate of metastatic melanoma is only 27% [5]. Immunotherapies like ipilimumab, nivolumab, and pembrolizumab are currently being used for the treatment of malignant melanoma and have shown better tolerance in patients as compared to earlier traditional cytokine-based treatment. In order to improve available treatments, researchers have been developing molecularly targeted therapies to target biomarkers responsible for melanoma progression and serve as an alternative or supplement to immunotherapy, which has revolutionized the treatment and management of malignant melanoma (Table 1). Recently, the combination of atezolizumab, vemurafenib, and cobimetinib have been approved by the FDA as first-line treatment for unresectable advanced BRAF V600 mutant melanoma and showed a significant increase in progression-free survival in patients [6].

**Figure 1:**
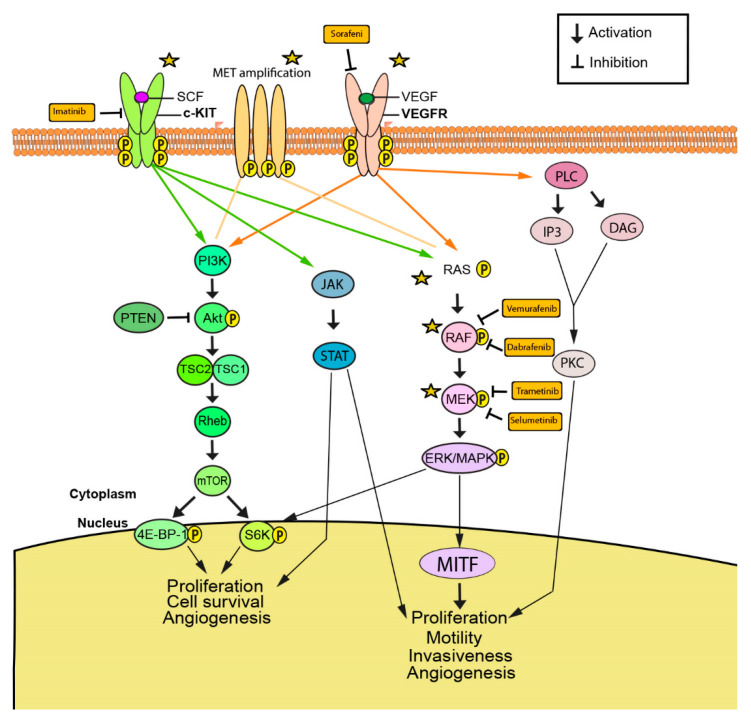
Predominant signaling pathways contributing to melanomagenesis and progression. Phosphorylation of receptor tyrosine kinases mesenchymal epithelial transition factor (c-MET), receptor-tyrosine kinase Kit (c-KIT) and vascular endothelial growth factor receptor (VEGFR) initiates signaling pathways resulting in proliferation, survival, motility, and angiogenesis. Activating mutations in downstream pathway components, such as reticular activating system (RAS), rapidly accelerated fibrosarcoma (RAF), and mitogen-activated protein kinase (MEK), confer constitutive pathway activation despite absence of growth signals [40,41,42]. Activation of epithelial mesenchymal transition (EMT) has been implicated in acquired resistance to multiple drugs that target aberrant signaling.

Currently, the following molecularly targeted therapies focus on genetic mutations in the following proteins: BRAF, MEK, members of the Ras family (NRAS, HRAS, and KRAS), c-KIT, c-Met, VEGFR, and PI3K (Figure 1). These mutations cause the activation of downstream pathways, such as RAS/MAPK, AKT/PI3K, and JAK/STAT, which affect cell proliferation and cell survival, causing cells to become tumorigenic (Figure 1) [40,41,42].

Small molecule inhibitors have been developed to treat melanoma, and numerous patients show promising results with these targeted treatments. Prolonged treatment by these small molecule inhibitors leads to acquired drug resistance, resulting in further progression of the disease [41,42]. For this reason, the issue of acquired drug-resistance has become a priority research area. Some modes of resistance are believed to be related to epithelial–mesenchymal transition (EMT) and the upregulation of various oncogenic proteins [43].

Various combinatorial therapies, such as different combinations of BRAF inhibitors with MEK inhibitors, have shown promise in delaying the onset of resistance, which would in turn increase the progression-free survival (PFS) and overall survival rates [44,45]. Targeted approaches have also proven useful as combinatory treatment with immunotherapy [46]. The synergistic effect of these therapeutic paradigms in malignant melanoma should be further elucidated by future research. The discovery of novel targets and strategies is imperative to overcoming acquired resistance to currently available molecularly targeted therapies in melanoma. This review will discuss various therapeutic targets and inhibitors used to treat melanoma, as well as the modes of resistance for these treatments and discuss their potential solutions.

## 2. BRAF

**Figure 2:**
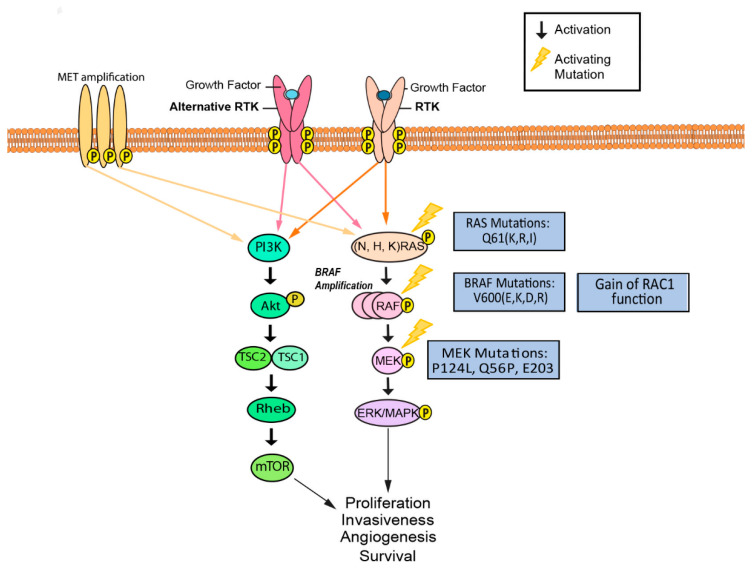
Activating mutations and activation of alternative upstream receptors confer resistance to inhibitors targeting aberrant MAPK signaling. Mutations in RAS, RAF, and MEK, as well as RAF amplification and activation of RAS-independent RAF variants, have been implicated in bypassing MAPK inhibition. Upregulation and activation of additional receptor tyrosine kinases may also contribute to enhanced MAPK signaling or alternative pathway activation, such as PI3K/Akt [56,57,58,59,60]. EMT has also been shown to contribute to BRAF inhibitor resistance.

BRAF is a serine/threonine-protein kinase that regulates cell proliferation, differentiation, and survival via the RAS/RAF/MAPK signaling pathway (Figure 2). BRAF relays and amplifies signals from RTK and RAS to downstream targets (Figure 2) [47,48]. As part of the RAF protein kinase family, BRAF possesses constant and regulatory structural domains that are critical to its regulatory role [49,50]. The constant domains include cysteine-rich CR1, serine/threonine-rich CR2, and CR3. The CR1 domain of BRAF interacts with GTP-bound RAS, inducing further activation of BRAF at the cell membrane [49]. Phosphorylation of the CR2 domain facilitates the localization and activation of BRAF, while CR3 serves as the catalytic kinase domain of BRAF when phosphorylated [49]. The catalytic site is in a cleft between the N-terminus lobe and the C-terminus lobe of the three-dimensional structure of BRAF [50]. BRAF is a treatment target in melanoma patients, as studies have found it to be mutated in over 60% of cutaneous melanomas [48,51]. Over 80% of BRAF mutations are caused by a V600E amino acid substitution, while the remaining 20% of mutations consist mostly of V600K and V600D substitutions (Figure 2) [48,52]. These missense mutations in the kinase domain of BRAF appear to cause changes in the interaction between the glycine-rich loop and the activation segment, conferring constitutive activation irrespective of RAS signaling [53,54]. Vemurafenib (PLX4720) was one of the first highly selective molecularly targeted inhibitors for mutant BRAF (V600E and V600K). Vemurafenib inhibits BRAF kinase activity by competing with ATP at the kinase domain, resulting in cell apoptosis and decreased tumor growth [7]. Dabrafenib is another ATP-competitive BRAF inhibitor shown to target all three V600 BRAF mutations [10]. Dabrafenib is often used in combination with trametinib, a MEK inhibitor and is FDA approved [55].

Although there are treatment options for patients with BRAF mutations, resistance commonly develops. Many mechanisms have been implicated in the resistance to BRAF inhibitors, each of which reactivates or bypasses the MAPK pathway after BRAF signaling has been inhibited. The possible routes of reactivation or bypassing of the MAPK pathway include: upregulation of platelet-derived growth factor receptor beta (PDGFRB); upregulation of insulin-like growth factor receptor I (IGF-1R); bypassing BRAF through overexpression of PI3K, COT kinase, or MEK mutations; presence of RAS-independent BRAF variants, mutated BRAF amplification signaling, amplification of microphthalmia-associated transcription factor (MITF); and loss of function of neurofibromin (NF1) [56,57,58,59,60,61]. Thus, therapeutics that inhibit BRAF and evade these mechanisms of resistance have gained significant interest as potential treatments of melanoma. Studies suggest that BRAF amplification in BRAF V600E mutants may lead to spontaneous dimerization of the resulting protein and reactivation of the ERK pathway [60,62]. It has been estimated that increased BRAF copy number is present in 20% of cases of acquired resistance to BRAFi therapy in melanoma [60]. Several MEK1 mutations have been observed in association with vemurafenib resistance in patient tumor samples, including MEK1 E203 and Q56 mutations [63]. Studies suggest that miR-1246, miR-204-5p, and miR-211-5p also serve as key players in the mechanism of acquired resistance [64,65]. Furthermore, there is evidence that MET, a hepatocyte growth factor and receptor kinase, is responsible for increasing resistance to BRAF inhibitors [66]. Finally, studies have also demonstrated that 50–60% of BRAFi-resistant melanoma cells possess activation of RhoA family GTPases. Hence inhibition of Rho and its downstream transcriptional effectors, MRTF and YAP1, should be explored for development of novel therapies [67]. RAC1 is a gene that codes for a GTPase and has been found to be mutated in up to 20% of patients no longer responding to BRAFi treatment [62]. These findings suggest that combination therapy targeting BRAF as well as one or more of these coactivated pathways may yield more favorable treatment results compared to targeting BRAF alone.

A vast array of therapeutics have been designed, tested, and modified as a result of these studies on the different mechanisms of resistance to BRAFi therapy. For example, two novel paradox BRAF inhibitors, PLX8394 and PLX7904, were designed in light of re-cent findings on BRAF amplification in acquired resistance and hold significant therapeutic potential. PLX8394 demonstrated efficacy in inhibiting V600-mutated oncogenic BRAF without activating MAPK simultaneously in colon adenocarcinoma and should be further explored in melanoma [12]. PLX8394 was also shown to reduce ERK1/2 reporter activity and induce apoptosis ex vivo in BRAF-mutant melanoma xenografts with greater efficacy than vemurafenib; however, studies in vivo demonstrated acquired resistance through ERK1/2 reactivation but with different ERK-inhibitor sensitivity [68]. Future studies should explore the framework of these paradox inhibitors to design a therapeutic that overcomes resistance caused by BRAF amplification in melanoma. In one study, treatment of BRAF-inhibitor (BRAFi)-resistant melanoma cells with a miR-1246 mimic demonstrated downregulation of p-ERK and reduced vemurafenib antiproliferative activity [65]. Overexpression of myeloid cell leukemia-1 (MCL-1) serves as another possible mechanism of resistance to either vemurafenib or dabrafenib individually or in combination with trametinib in melanoma cells [69]. MCL-1 is a pro-survival member of Bcl-2, which is known to inhibit apoptosis and promote oncogenesis, leading to immortalization of malignant cells [70]. One study found high levels of MCL-1 in cell lines resistant to BRAF/MEK inhibitors, and addition of an MCL-1 inhibitor to that combination resulted in reduced cell growth, increased cell death, and a delay in resistance [71]. A functional genomics study conducted using genome-scale open reading frame (ORF) resistance screens revealed a cyclic AMP-dependent melanocytic signaling network as another possible contributor to BRAF/MEK inhibitor resistance in melanoma [72].

MITF is a master lineage transcription factor that plays a crucial role in melanocyte development, and increased copy number of MITF has been associated with BRAFi resistance through MAPK pathway alterations [73,74]. Upregulation of MITF has been correlated to late stage disease states and poor patient outcomes, so MITF suppression is a therapeutic pathway worthy of exploration [75]. Direct targeting of MITF with small molecule inhibitors has proven to be a challenge; however, indirect suppression of MITF through histone deacetylase inhibitors has been shown to reduce MITF mRNA expression in melanoma [76]. CH5552074 and CH6868398 are two MITF protein suppressors described by Aida et al. that work through this alternative mechanism to inhibit MITF amplification, as often seen in metastatic melanoma [76]. CH6868398 displayed in vivo efficacy in a melanoma xenograft model, and combinatory treatment with PLX4720 BRAFi therapy induced apoptosis in BRAF-mutated melanoma cells [76]. MITF is also an important player in the study of combined targeted and immunotherapy approaches. In both preclinical models and patients, the upregulation of MITF after BRAFi therapy has been correlated with simultaneous upregulation of melanocyte lineage antigens such as tyrosinase [72,77]. These may serve as antigens to tumor-infiltrating CD8+ T cells and play a key role in the elimination of melanoma cells [77,78]. Further studies focused on identifying and developing specific T-cell clones that display tumor specificity should thus be conducted, as these would serve as a complementary approach to BRAFi therapy [77].

RhoA family GTPases may serve as additional therapeutic targets [79]. RhoA activation occurs in 50–60% of melanoma cell lines resistant to vemurafenib, likely as a result of transforming growth factor beta (TGF-β) [67]. The role RhoA plays in resistance is complex and is not entirely understood; however, it may modulate ERK function and act through a downstream effector known as MRTF [67]. The use of an inhibitor of Rho kinase (ROCK), which is modulated by RhoA, has been shown to resensitize melanoma cell lines to BRAF inhibitors [67]. RhoA may also act through downstream effectors like YAP1, which has already been shown to confer resistance to BRAF/MEK inhibitor treatment in melanoma [80]. Future studies should explore the mechanisms through which RhoA confers resistance. Insulin-like growth factors, including insulin-like growth factor 2 mRNA binding protein (IGF2BP1), have also demonstrated ability to confer resistance to tyrosine kinase inhibitors (TKIs). IGF2BP1 inhibition increased the efficacy of BRAF and BRAF/MEK inhibitors in V600E-mutant melanoma, suggesting that this protein may serve as a therapeutic target [81]. E2F1, a transcription factor with diverse roles in apoptosis and the cell cycle, is upregulated in BRAFi-resistant melanoma and increases the expression of insulin-like growth factor 1 receptor (IGF-1R) [82]. This upregulation likely occurs due to PI3K/AKT signaling activity, and targeting both E2F1 and IGF-1R may help to combat resistance in melanoma [82]. Neurofibromin is another GTPase-activating protein encoded by the NF1 gene that regulates cell differentiation and proliferation [62]. Since NF1 serves as a negative regulator of RAS, the first protein in the MAPK signaling cascade, mutations that cause a loss of NF1 function have been implicated in constitutive MAPK activation and acquired resistance to BRAFi therapy in melanoma [62,83]. NF1 mutations were found in up to 17% of samples in the Melanoma Genome Project report [84].

Glucocorticoids are novel hormones and have gained interest as therapeutic option for melanoma as they play a complicated role in melanoma and other cancers. Past studies found that glucocorticoid receptor (GCR) knockdown inhibited synthesis of glutathione, a stress hormone known to regulate metastatic growth in aggressive B16-F10 melanoma cells [85]. Recent studies by Estrela et al. discovered that antagonizing glucocorticoid receptors may overcome BRAFi resistance in melanoma [13]. This novel investigation used glucocorticoids in mice inoculated with BRAF V600E melanoma and found that the steroid addition overcame BRAFi resistance [13]. These findings may seem counterintuitive since glucocorticoids are sometimes used as an adjunctive to treatment. While therapeutic doses of glucocorticoids may increase apoptosis of cancer cells, physiologic levels may actually contribute to tumor survival, likely through AKT and NF-κB signaling [13]. Aryl hydrocarbon receptor (AhR) is another recently implicated target. AhR is constitutively activated in small subpopulations of melanoma cells that are likely responsible for relapses after BRAFi treatment [86]. AhR binds its ligands and relocates to the nucleus where it acts as a transcription factor. Antagonizing AhR with vemurafenib moderately decreased tumor growth when compared to vemurafenib alone [86]. Researchers have also studied the role of FGF1 in developing resistance to BRAF/MEK inhibitors. One study found this growth factor to be upregulated in melanoma cell lines resistant to BRAF and MEK inhibitors, activation of the FGFR cascade leads to sustained ERK function. Furthermore, adding FGF1 inhibitors to the combination may resensitize melanomas to BRAF/MEK inhibitors [87].

In summary, the possible mechanisms of resistance in melanoma to both solo BRAFi therapy and combinatory BRAF/MEK inhibitor treatment include a wide variety of parallel pathways, cell signaling, and transcription patterns. These factors contribute to the difficulty of treating melanoma, despite the knowledge that V600E most often serves as the driving mutation [48]. Furthermore, the combination of BRAF inhibition with different immunotherapeutic approaches like immune checkpoint inhibitors, vaccine, adoptive T cell therapy, and cytokines IL-2 and α-2b/peg-IFN have been the focal point of a number of clinical trials, demonstrating the importance of this area of study [46]. Effective treatment will require continued innovation by researchers to discover resistance pathways and develop therapies to overcome them. Additionally, personalizing care to ensure that each patient receives treatment best suited to their subset of cancer cells will be imperative in providing good outcomes.

## 3. MEK

Mitogen-activated protein (MEK)/extracellular-signal regulated kinase (ERK) 1 and 2 are homologs of a dual-specificity kinase (DSK) that are capable of phosphorylating both threonine and tyrosine residues on target proteins [88,89]. RAF phosphorylation and activation of MEK1/2 by RAF leads to phosphorylation and activation of ERK1/2 (Figure 2) [90,91]. Activated ERK1/2 then mediates regulation of secondary targets involved in survival, differentiation, and proliferation. This makes MEK1/2 an ideal therapeutic target in melanoma, as these pathways are often mutated [92].

A number of MEK inhibitors, including selumetinib, trametinib, cobimetinib, and binimetinib, have been studied as monotherapy or in combination with BRAF inhibitors for the treatment of melanoma. The combination of trametinib with dabrafenib was approved for use against BRAF V600E/K mutations and improved survival rates over dabrafenib alone with no increased toxicity [11,93]. More recently, vemurafenib with cobimetinib and encorafenib with binimetinib have also been FDA approved as BRAF/MEK combination therapy for the treatment of melanoma [14,15]. Further research found that combining a MEK inhibitor, binimetinib, with a BRAF inhibitor, encorafenib, as opposed to a BRAF inhibitor alone, yielded long term benefit in progression free survival in patients with melanoma [94]. Another therapy recently approved by the FDA is the combination of atezolizumab with BRAF inhibitor vemurafenib and MEK inhibitor cobimetinib, resulted in safe tolerability and prolonged progression-free cell survival in melanoma patients [6]. Additional trials are currently being conducted to assess the efficacy of MEK inhibitors used in combination with BRAF and/or PD-1 or PD-L1 inhibitors. One neoadjuvant trial using dabrafenib, trametinib and/or pembrolizumab has shown that these treatments are well tolerated and demonstrate efficacy in treating BRAF-mutated stage III melanoma [77]. Selumetinib, a MEK1/2 inhibitor, showed potential when used in combination with chemotherapeutic dacarbazine [16]. Treatment with selumetinib alone was only effective in cells expressing low levels of pAKT, suggesting that the PI3/AKT pathway may be responsible for resistance to this MEK inhibitor. Trials coupling selumetinib with AKT inhibitor MK-2206 did not yield promising results, possibly due to drug toxicity, preventing minimal effective dosing [14]. One novel MEK-RAF inhibitor, labeled CH5126766, has previously been shown to inhibit various solid tumors. However, its utilization has been halted by its toxicity. A recent phase I study testing various drug dosages and frequencies of CH5126766 found it to be effective in halting cancers with RAF-RAS-MEK mutations, including melanoma. Further evaluation of this novel inhibitor in combination with other anti-cancer agents is needed [17]. CK2α is an additional serine/threonine kinase involved in cell growth and apoptosis that has also been found to be elevated in melanoma cell lines. CK2α was shown to decrease the efficacy of dabrafenib in BRAF-mutant melanoma cells via its kinase-independent scaffolding function [95].

The use of MEK inhibitors as primary therapy presents complications due to numerous mechanisms of resistance to these inhibitors. Amplification of BRAF, an upstream signaling component, leads to hyperactivation of MEK and decreases the ability of inhibitors to exert their effects on MEK-mediated ERK phosphorylation [96]. BRAF amplification demonstrates another potential mechanism of K-Ras activation which provides tumor cells with the ability to overcome their sensitivity to MEK inhibition [97]. Clinical data suggests that patients with continued disease progression after treatment with a RAF inhibitor have a lesser likelihood of benefiting from MEK inhibitors [73] MEK1 mutations such as P124L and Q56P, also confer resistance to therapies with both MEK (AZD6244) and BRAF inhibitors (PLX4720) through direct interference of drug binding or conformational changes that disrupt the drug binding location (Figure 2) [63]. In addition, activation of the STAT3 pathway has been associated with MEK inhibitor resistance through impairment of Bcl-2-like protein 11 (BIM), a member of the Bcl-2 family that is required for tumor suppression [98]. Increased expression of the immunoglobulin transcription factor 2 (ITF-2) gene, which codes for a transcription factor involved with lymphocyte development, has also been implicated as a mechanism of acquired MEK inhibitor resistance [99]. ITF-2 transcription is targeted by the Wnt signaling pathway, and ITF-2 expression was found to be significantly upregulated in MEK inhibitor-resistant melanoma cell lines [99]. Subsequent knockdown of ITF-2 resulted in increased sensitivity of resistant cells to selumetinib [99]. Activation of Wnt signaling via p-ERK and GSK3β has also been found in resistant cells [99]. Additionally, regulation of extracellular matrix proteins can also confer resistance to MEK inhibitors. A previous imaging study found that trametinib-resistant melanoma cells required close proximity to bundled collagen early in the process of resistance development; however, as resistance to the drug became more established in the tumor, this association was no longer seen [100]. Stiffening of tumors due to deposition of collagen and tumor reorganization may promote MEK inhibitor resistance [100]. Furthermore, downregulation of a protein encoding gene, DUSP4, has been shown to contribute to MEK resistance in BRAF wild-type melanoma [101].

In some cases, dual resistance to both BRAF and MEK inhibitors can arise and present a significant treatment challenge. The p90 subfamily of ribosomal S6 kinase (p90RSK) of kinases may serve as a target with the potential to bypass this dual resistance. RSK inhibition with BI-D1870 and BRD7389 resulted in a significant reduction of protein synthesis and proliferation in dual-resistant melanoma cell lines to BRAF and MEK inhibitors [102]. Similarly, synergism between immune checkpoint inhibitors (ICIs) with BRAF and MEK inhibitors as a combination therapy holds promise, as the two approaches have shown complementary activity. For example, combination therapy with pembrolizumab, which is a PD-1 inhibitor, along with dabrafenib and trametinib decreases the occurrence of resistance [14]. At the same time, resistance to this triple therapy is still noted. Another study utilizing longitudinal whole exome sequencing revealed treatment failure after mutations were acquired during treatment to MAP2K2, a gene encoding for MEK1, as well as beta-2-microglobulin, a protein with a role in antigen presentation [103]. Furthermore, the IMspire150 trial conducted in 2020 demonstrated statistically significant improvement in progression-free survival from 10.6 months to 15.1 months in advanced untreated melanoma patients treated with vemurafenib, cobimetinib, and checkpoint inhibitor atezolizumab instead of vemurafenib, cobimetinib, and a placebo [62].

Additional research suggests that resistance to MEK inhibitors and other melanoma therapies involved in this pathway, including BRAF inhibitors, are complex and intricately related. Analysis of melanoma specimens suggests that tumors are made up of heterogeneous groups of cells with a diversity of often reversible epigenetic changes, genetic mutations, and transcriptional regulators that allow cancer to escape eradication by treatment. Tumor variability within a patient and melanoma plasticity may contribute to the difficulty in treating melanoma with TKIs [104].

## 4. NRAS, HRAS, KRAS

The RAS subfamily is part of the 5′ GTP-binding protein family, and is composed of NRAS, HRAS, and KRAS, all of which are expressed in human cancer cells (Figure 2). Due to their appearance in an estimated 20–30% of all human cancers, RAS mutations are a frequently sought-after target for cancer therapies [105,106]. Additionally, NRAS mutations have been found in 15–20% of melanoma cases [107]. KRAS, an EGFR-induced cell signaling downstream mediator, has been found to be mutated in about 25% of Non-small cell lung cancer (NSCLC) cases, whereas in melanoma, KRAS mutations are rare and found in only 1.7% of the cases [108,109]. One clinical study compared prognostic significance between a cohort of BRAF-mutant and NRAS-mutant melanoma patients, revealing a lower relative survival in patients with NRAS-mutant melanoma. [110,111]. NRAS is mutated in 15–30% of cutaneous melanoma, and patients having NRAS mutations in melanoma are generally older (>55 years), have thicker tumors, and have inferior clinical outcomes in comparison to patients harboring BRAF mutations [108]. RAS is a target for cancer therapy due to its involvement in multiple cell signaling pathways that contribute to apoptosis, proliferation, and differentiation. RAS can affect the MAPK pathway through phosphorylation of MEK1 and MEK2, which activate ERK1 and ERK2, leading to cell-cycle progression (Figure 2) [112,113]. Furthermore, RAS can affect the PI3K/ALK pathway through interaction with the phosphatidylinositol 3-kinase (PI3K) subunit and directly contribute to anti-apoptotic activity [112].

While RAS is involved in multiple signaling pathways and its mutations have been extensively studied, direct inhibitors are yet to be found. For example, downstream ERK activity by the mechanisms that cause increased RAF dimerization and upregulation of ERK signaling can compensate for RAS inhibition [57]. In addition, increased activity of parallel growth factor receptors, such as PDGFRβ, IGF1R and EGFR, can induce RAS activity, resulting in resistance to the inhibitors [57]. Furthermore, MAP3K8, which encodes a serine-threonine kinase called COT/Tpl2, has been identified as a MAPK pathway agonist that causes resistance to RAF inhibition [114]. COT bypasses RAF signaling by activating ERK through MEK-dependent mechanisms. One study found that the loss of NF1, which frequently co-occurs with RAS alterations in melanoma, is associated with loss of negative feedback to RAS activation, resulting in increased activation of RAS and downstream resistance to RAF inhibition [61]. In addition, mutation of functional NF1 is associated with BRAFi resistance in melanoma and mutations in the PTPN11, SOS1, RAF1 and SPRED1 genes frequently co-occur with NF1 loss [62]. As a result of these mechanisms, current targeted therapies focus on interference with downstream pathways of mutant RAS proteins, such as the MAPK and PI3K pathways. A phase II clinical trial demonstrated that one MAPK downstream protein, a MEK inhibitor named MEK 162, was able to achieve a 21% response rate in NRAS-mutated patients [115,116]. These promising results show MEK162 to be an active targeted approach for NRAS mutations in patients with melanoma. Another study revealed that combined inhibition of MEK and mitotic regulator Polo-like kinase 1 (Plk1) resulted in significant growth reduction of NRAS-mutant melanoma cells in vitro and also regression of xenografted NRAS-mutant melanoma in vivo [117]. An additional experiment studied the combinatory inhibition of MEK and pan-RAF, which are both downstream signaling proteins in the MAPK pathway, and showed significant growth inhibition in NRAS-mutated human melanoma cell lines [118].

Several next generation treatment efforts have searched for downstream inhibitors and combination therapies that achieve RAS inhibition and some next-generation treatments target MEK inhibition. For example, CH5126766/RO5126766 is a dual RAF/MEK inhibitor that shows potency and selectiveness in clinical trials. In both BRAF-mutated and NRAS-mutated melanoma cell lines, CH5126766/RO5126766 were shown to arrest the cell cycle in G1; meanwhile, SK-MEL-2 xenografts demonstrated suppression of tumor growth [18]. Further clinical trials have investigated the therapeutic use of two additional MEK inhibitors under investigation for NRAS-mutant melanomas. Binimetinib and pimasertib are allosteric MEK inhibitors under phase II and III trials that have demonstrated anti-cancer effects and may be promising treatment options. A previous phase II study of binimetinib revealed that 20% of advanced NRAS-mutant melanoma patients generated a partial response to treatment, and 43.3% displayed stable disease [115]. As a result, binimetinib is undergoing phase III clinical trials comparing its efficacy against dacarbazine in unresectable/metastatic-mutant NRAS melanoma patients [119,120]. Pimasertib (AS703026) showed selective antiproliferative effects on tumor cells with high MEK-ERK1/2 activity [19,121]. Due to favorable results in phase I trials, pimasertib is the subject of a phase II trial comparing its efficacy to dacarbazine in NRAS-mutant cutaneous melanoma [20]. Downstream NRAS inhibition combination therapy may prove to be an effective therapy for patients suffering from melanoma. Future trials can elucidate support for the growing evidence of effectiveness of such targeted therapies.

## 5. c-KIT

**Figure 3:**
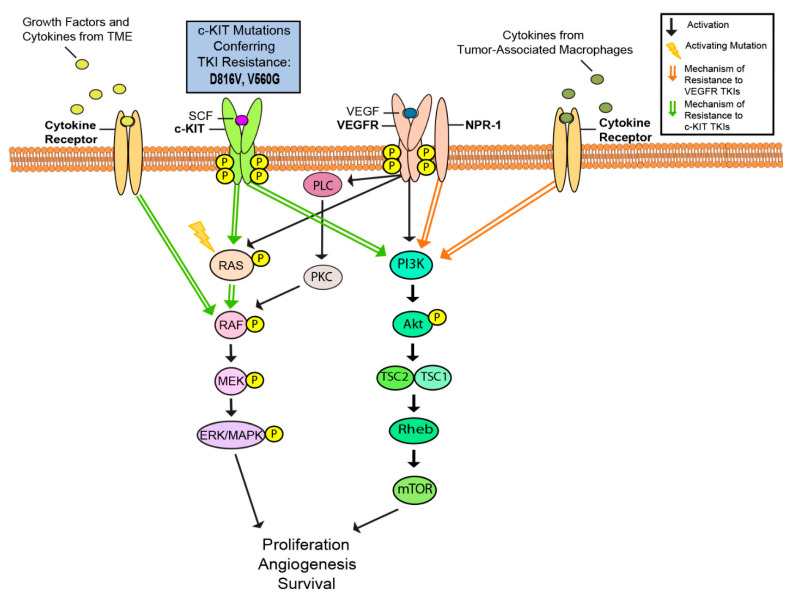
MAPK, PI3K, and angiogenic reactivation confers resistance to c-KIT and VEGFR TKIs [1]. Activating c-KIT mutations and pathway reactivation by growth signals from the tumor microenvironment (TME) contribute to resistance to c-KIT TKIs [129,130]. Angiogenic signaling from tumor-associated macrophages as well as pathway reactivation via VEGFR co-receptor NRP-1 may contribute VEGFR TKI resistance in melanoma [136,137,138].

c-KIT is a type III transmembrane receptor tyrosine kinase (RTK) found on the surface of cells in many cancer types, including gastrointestinal stromal tumors (GIST), systemic mastocytosis, and subsets of acute myeloid leukemia and melanoma [122,123,124,125,126]. c-KIT binds to stem-cell factor (SCF), a glycoprotein ligand, and undergoes receptor dimerization, autophosphorylation, and activation of intrinsic tyrosine kinase activity [127,128]. Subsequent binding of intracellular signaling proteins initiates downstream signaling of the MAPK, PI3K-Akt, and JAK-STAT pathways, leading to specific responses relating to cell proliferation and survival (Figure 3) [1]. Activating mutations in c-KIT are identified in approximately 15.6% of mucosal melanomas and 23% of acral melanomas and these numbers rises up to 39% and 36% respectively when KIT copy number increases [129,130]. c-KIT mutations are most commonly found in exons 9 and 11 [131]. Mutations in c-KIT can either activate or deactivate the receptor, causing varying downstream functional consequences in different cancers. For instance, in metastatic melanoma, activation of c-KIT causes apoptosis, while activation in uveal melanoma results in increased proliferation [132,133,134]. Imatinib is a selective RTK inhibitor that binds the kinase domain of c-KIT. Inhibition of c-KIT-mutant melanoma with imatinib has produced objective response rate of 24.4% and disease control rate of 66.7% in patients with mutations in exons 11 (L576P) and 13 (K642E) [21]. In comparison, treatment of c-KIT-mutant GIST with imatinib yielded a response rate of 80%, with over 90% of patients remaining progression free at one year [22,135]. Future studies should explore the discrepancy in response rates between c-KIT inhibition in c-KIT-mutant GIST and melanoma.

An approach conjugating imatinib to an antibody targeting c-KIT should be explored in melanoma cells, as this strategy has exhibited enhanced efficacy in several cancer types [139]. Diminished clinical efficacy of imatinib therapy in c-KIT mutant melanoma may be a result of secondary acquired resistance caused by higher genetic mutation load, presence of activating or secondary c-KIT mutations, re-activation of redundant downstream survival signaling pathways, and/or tumor microenvironment cytokines [140,141,142,143,144]. Newer c- KIT TKIs, such as masitinib, sunitinib, dasatanib, and nilotinib, have shown modest efficacy, most notably in patients with tumors already resistant to imatinib [21,26,145]. A previous study found that masitinib caused tumor regression after brain metastasis in patients with c-KIT-mutant esophageal melanoma [146]. These treatment options should be studied further to better understand the cause of resistance [147].

Due to extensive UV damage, the mutation load in melanoma is high compared to other tumor types [148]. The average somatic mutation rate of c-KIT-mutant melanomas is even higher (30 mutations per Mb, *n* = 3, *p* = 0.02) compared to severely sun-damaged (SSD) skin (21 per Mb) and non-SSD skin (3.8 per Mb) [148]. Mutations in c-KIT have been found in up to 28% of melanomas on chronically sun-damaged skin, but not in non-acral melanomas, unrelated to chronically sun-damaged skin [21]. In some cases of melanoma, resistance to targeted therapy appears to be related to the acquisition of new mutations in other genes that contribute to tumor growth. For example, a previous study found that an acquired activating N-RAS mutation was associated with c-KIT inhibitor resistance in c-KIT-mutant melanoma [149]. Moreover, in c-KIT-mutant acral melanoma, the addition of TKIs targeting MET and KIT showed increased efficacy compared to KIT alone in the presence of hepatocyte growth factor, the ligand for MET [150]. In addition to the higher mutation load, the presence of activating mutations contributes to imatinib resistance in c-KIT-mutant melanoma. Imatinib is less effective in treating melanoma with activating mutations in the c-KIT kinase domains compared to those with activating mutations in the juxtamembrane (JM) domain (encoded in exon 11), which is known to have an autoinhibitory function [151]. An S628N substitution in exon 13 was identified as a gain-of-function mutation, and melanoma carrying this c-KIT mutation demonstrated susceptibility to imatinib treatment [152]. However, mutations in D816V and V560G **conferred** acquired resistance via activating mutations [153,154,155]. Another mechanism of resistance arises from downstream signaling pathways of c-KIT, through either ligand binding, stem-cell factor (SCF), or an oncogenic mutation. c-KIT has been shown to drive melanocyte proliferation and melanoma survival through activation of the phosphatidylinositol 3-kinase (PI3K) and mitogen-activated protein kinase (MAPK) pathways [142,143]. Stimulation of PI3K is required for full MAPK activity in response to c-KIT, suggesting that PI3K signaling is the dominant effector of c-KIT-mediated proliferation and survival in c-KIT-mutant melanomas [143]. Due to simultaneous reactivation of MAPK function, selective PI3K inhibition did not replicate imatinib activity in c-KIT-mutant melanoma. However, combinatory inhibition of both PI3K and MAPK pathways yielded promising results [143]. These findings highlight the central role of targeting PI3K/MAPK cascades in the treatment of c-KIT mutant melanoma. Another downstream target of c-KIT, lemur tyrosine kinase 3 (LMTK3), has also shown promise. In cell lines and mice, siRNA silencing of the gene for this kinase led to cell death in c-KIT-mutated melanomas and GIST tumors, even those with drug resistance, without effecting cells not dependent on c-KIT [156].

Finally, imatinib resistance can also develop at the tumor microenvironment level. Tumor cells interact with surrounding endothelial and stromal cells, as well as growth factors and cytokines secreted by these cells, which may reduce the sensitivity of tumor cells to imatinib [157]. In a study investigating c-KIT-positive metastatic uveal melanoma (UM), increased imatinib resistance was observed in cells incubated with either SCF-supplemented medium or microvascular endothelial cells-conditioned medium [144]. The addition of exogenous SCF in culture medium of UM cell lines did not stimulate proliferation, but instead caused a significant reduction in the inhibitory effects of imatinib in c-KIT-positive UM [144]. It is hypothesized that SCF ligand binding to c-KIT causes conformational changes in the ligand-mediated receptor dimerization complex, preventing stable binding of imatinib. Additionally, previous X-ray crystallographic studies have shown that imatinib can bind to the inactive conformation of c-KIT, further validating this hypothesis [151]. FGF2 is a growth factor shown to induce resistance to nilotinib, a **second-generation** drug of the imatinib family, likely through activation of the MAPK pathway [158,159]. Ponatinib is a multi-target TKI previously approved to treat chronic myeloid leukemia (CML) caused by BCR-ABL fusion. It has also been shown to have activity against both c-KIT and FGF2, and it has demonstrated promise in treating c-KIT-mutant melanoma, warranting further study [160,161].

c-KIT-mutant melanoma is likely to develop resistance to molecular-targeted therapies due to pre-existing genetic alterations, reactivation of downstream c-KIT signaling pathways, and dynamic interactions with the microenvironment. Current management of c-KIT mutant melanoma involves clinical trials using a c-KIT inhibitor or immunotherapy, as there is currently minimal data for the efficacy of immune-modulators in c-KIT mutant melanomas [140]. Combinatorial therapies simultaneously targeting multiple pathways such as PI3K/MAPK, growth factors that may confer resistance, and immunotherapies should be explored in the setting of treating naïve c-KIT-mutant melanoma and patients with acquired resistance to c-KIT inhibitors.

## 6. VEGFR

Angiogenesis is a key characteristic of tumor expansion. Vascular endothelial growth factor (VEGF) is an essential mediator promoting angiogenesis shown to be upregulated in melanoma and a number of other cancer types [162,163,164,165,166]. Previous studies have suggested that VEGF plays an important role in tumor extravasation and immune evasion through activation of the PLC/PKC/MAPK pathway and by promoting endothelial cells to secrete prothrombic mediators like von Willebrand factor (vWF) and P-selectin (Figure 3) [167,168]. In addition, malignant cells produced higher amounts of VEGF compared to normal cells [169]. In vitro stimulation of VEGFR-2 by VEGF-A resulted in increased proliferation, suggesting an autocrine interaction between tumor-derived VEGF-A and VEGFR-2, promoting survival and proliferation [169].

The therapeutic inhibition of VEGF ligand and/or receptor is an approach with notable clinical potential. Sorafenib is a multi-kinase inhibitor against VEGFR-1, VEGFR-2, VEGFR-3, BRAF, and c-Kit that downregulates both angiogenesis and tumor proliferation in multiple cancer cell lines [27,170,171]. Axitinib is a selective inhibitor against VEGFR-1, VEGFR-2, and VEGFR-3 that competitively binds to the receptor’s ATP-binding domain, compromising its activity [172]. Studies have shown axitinib to have an antitumor effect both in vitro in murine melanoma cells and in vivo melanoma xenografts, as well as in melanoma patients when used in combination with toripalimab, an anti-PD-1 monoclonal antibody [28,29]. Furthermore, bevacizumab, a monoclonal antibody against VEGF-A has demonstrated clinical efficacy in the treatment of a number of cancers, including melanoma, both as a stand-alone drug and in combination with chemotherapeutics like carboplatin and paclitaxel [173,174,175]. The clinical efficacy of bevacizumab in combination with other immunotherapies, such as ipilimumab and atezolizumab, is also being explored [176,177]. After favorable phase I results patients with late-stage melanoma are undergoing a phase II clinical trial with bevacizumab coupled with ipilimumab, a monoclonal antibody against CTLA-4 [176]. Another monoclonal antibody, D16F7, works against VEGFR-1 and has been shown to reduce in vivo angiogenic activity in a matrigel plug assay, as well as decreased tumor growth in mice bearing B16F10 melanoma cell tumors [178]. DW10075 is another selective inhibitor of VEGFR-1, VEGFR-2, and VEGFR-3. A recent study demonstrated reduced cell proliferation and tumor growth in A375 melanoma cells following treatment with DW10075 via abrogation of VEGFR downstream signaling. However, acquired drug resistance needs further investigation [30].

Multiple mechanisms have been proposed to contribute to acquired VEGFR inhibitor resistance. In one study, high baseline plasma levels of VEGF-A and reduced expression of neuropilin-1 (NRP-1), a co-receptor of VEGFR, were associated with improved survival in patients treated with bevacizumab [179]. Studies suggest that VEGF-C levels are also affected by bevacizumab, as it allows for the sustained activation of VEGFR-2 and tumor growth [138]. Previous studies demonstrated that PDGF-C, a platelet derived growth factor, binds directly with NRP-1 in melanoma cells, while also stimulating extracellular matrix invasion and p130C phosphorylation in melanoma cells lacking PDGFRα [180]. Inhibition of PDGF receptor signaling sensitizes cells to anti-VEGF/VEGFR treatment, suggesting a possible route of resistance and a potential therapeutic target in anti-VEGFR melanoma therapy [181]. Another study showed mRNA expression of VEGFR-1, VEGFR-2 and VEGFR-3 to be lower in cell lines resistant to VEGFR-2 TKI, suggesting that avoidance of VEGFR-2 signaling dependency may serve as a mechanism of resistance [182]. Alternatively, an increase in the TKI sorafenib resulted in lysosomal uptake and sequestration, conferring resistance in tumor cells, and may be avoided if used in combination with verteporfin, a photosensitizer [183]. Furthermore, pro-angiogenic expression of VEGF-A in melanoma is activated by SRSF1, a pre-mRNA splicing factor, through phosphorylation of SRPK1 [184]. Blocking the pro-angiogenic VEGF-A_xxx_ isoform by inhibiting SRSF1 phosphorylation by SRPK1 inhibitors has been shown to reduce melanoma proliferation [184]. The use of SRPKIN-1, an irreversible SPRK1/2 inhibitor, was effective at increasing the anti-angiogenic isoform VEGF-A_165_b [185]. Additionally, VEGF_xxx_b isoforms inhibits the angiogenic activity of VEGF and may be effective in anti-cancer therapy [186]. In a study using VEGFR2-Fc-resistant murine melanoma models, the FGF2 signaling pathway was found to play a key role between endothelial cells and pericytes in maintaining tumor vasculature in acquired anti-VEGF-resistant tumors [187].

Tumor associated macrophages (TAMs) have also been implicated in the development of resistance to VEGF. Studies suggest that anti-VEGF agents create a hypoxic environment that attracts macrophages, which secrete angiogenic compounds, such as FGF-1/2, MMP9, and Ang2 [136,137,188]. Macrophages within the tumor play a dynamic role where they influence both susceptibility and resistance to anti-VEGF treatment [136,189]. One study found that mice treated with a colony stimulating factor inhibitor AC708, an anti-VEGF antibody, and paclitaxel had 83% lower tumor burden at the end of the study than mice who were treated with these drugs alone, suggesting that prevention of macrophage differentiation and proliferation may inhibit development of resistance to these drugs [190]. Another study found that the therapeutic use of the anti-VEGF antibody D16F7 prevented chemotaxis of M2 macrophages to melanoma tumor, increasing the efficacy of ICIs [191]. Additionally, it has been proposed that D16F7 could be used in VEGFR-1 positive melanoma cells with resistance to vemurafenib [192]. Recent studies have shown that rapamycin (also called sirolimus), an mTOR inhibitor, blocks the mTOR signaling pathway leading to suppression of angiogenesis and lymphangiogenesis in melanoma, and downregulates the expression of VEGF-A/VEGFR-2 and VEGF-C/VEGFR-3. Hence, rapamycin can be used as a targeted therapy against mTOR signaling pathway to prolong patient survival time [193]. Further research is needed to better understand the role of TAMs and anti-VEGF agents in anti-cancer therapy.

Despite a number of studies dedicated to VEGF- and VEGFR-targeting therapies, this approach has not yet translated into improved outcomes for melanoma patients. Studies have been conducted for the improvement of currently available therapies, such as sorafenib and gefitinib [194,195]. Quinazoline and thiourea-containing sorafenib analogs have been developed as dual EGFR and VEGFR-2 TKIs and have demonstrated promising anti-tumor activity in melanoma xenografts [194]. The development of novel therapies is crucial for improving treatment of this aggressive disease [194]. Given the crucial role of VEGF in tumor growth and metastasis, additional research is necessary to improve the efficacy of current anti-VEGF therapies, as well as to further elucidate the role of VEGF in extracellular matrix remodeling, cell migration, invasion, and inhibition of immune responses [196].

## 7. c-MET

c-Met is a receptor tyrosine kinase that binds with its ligand, hepatocyte growth factor (HGF), and leads to activation of the MAPK and PI3K/Akt pathways, resulting in growth, angiogenesis, invasion, and metastasis [91,197,198,199,200,201]. The MET gene in melanoma is regulated by PAX3, SOX10 and MITF [202]. c-MET is physiologically relevant for regulating cell motility via disruption of cell–cell interaction, such as in wound repair and embryogenesis [203]. c-Met expression also plays a role in the development of various types of cancers, including melanoma [198]. Overexpression of c-Met may be accomplished through the action of transcription factors PAX3 and ETS1, and these factors can be targets to repress melanoma tumor growth [204]. Normal melanocytes express c-Met and are receptive to HGF; however, melanoma cells produce HGF, creating an autocrine loop that constantly activates the c-MET receptor [198]. The level of c-Met expression in melanoma may be correlated with the degree of malignancy and prognosis [205]. c-Met activation is associated with angiogenesis in cancer, involving crosstalk with RTKs such as epidermal growth factor receptor (EGFR), insulin-like growth factor receptor (IGFR), and erb-b2 receptor tyrosine kinase (ERBB2) [206,207]. A multitude of studies have been conducted on drugs that inhibit c-Met in melanoma [208]. Initially, combinatory therapy like sorafenib/tivantinib and cabozantinib/vemurafenib were used as potential therapeutic strategies for targeting c-Met-mutant melanoma [209]. These combinatory therapies are important, as HGF/c-Met signaling has been implicated in the conferral of resistance to BRAF and MEK inhibitors, and c-Met inhibitors hold promise as potential additions to these therapies to promote tumor regression [66]. Unfortunately, as with most TKIs, the development of resistance to c-Met inhibitors is a major concern [210].

Inhibitors like savolitinib are type-I inhibitors that are highly selective for c-MET compared to other kinases [211]. The specificity of these inhibitors is due to a π-stacking interaction between the inhibitor aromatic groups and a tyrosine residue (Y1230) present in the activation loop (A-loop) of the kinase. A point mutation in c-MET causes disruption of these stacking interactions and has been observed in patients treated with c-MET selective type-I compounds such as crizotinib and savolitinib [211,212]. The cause of c-Met inhibitor resistance is still unclear, but studies have proposed many possible mechanisms. One study found that c-Met TKI resistance may be mediated by MET gene amplification rather than mutation, which leads to increased MET expression and amplification of KRAS, resulting in MAPK activation independent of c-Met [213]. Another study identified two resistance mechanisms to c-Met inhibitors using a gastric carcinoma cell line. One possible mechanism involves an Y1230 mutation in the c-Met activation loop, preventing the auto-inhibitory conformational change of c-Met [214]. Another proposed mechanism suggests overexpression of TGF-α leads to upregulation of epidermal growth factor receptor (EGFR) and activation of downstream signals that are also activated by c-Met [214]. These mechanisms may apply to TKI resistance in melanoma as well. Additionally, studies have found that c-Met TKI resistance might be due to upregulation of alternative cellular pathways, including Wnt and Akt/mTOR pathways [40]. This finding suggests that there is potential for combination therapy involving mTOR, Wnt, and c-Met inhibitors [40]. Furthermore, a study involving pathway inhibition of gastric cancer cells has demonstrated a greater rate of gastric cancer cell death when inhibition of c-Met and the MEK-ERK pathway are combined, providing an additional potential combinatorial therapy to counter resistance to c-Met inhibition in melanoma [215]. Further studies on combination therapies can prove to be the promising approach to ensure the better survival rate in patients.

## 8. PI3K/AKT

Genomic alterations predicted to activate the PI3K/AKT pathway have been detected in approximately 50% of all molecular subtypes of cutaneous melanoma [216]. PI3K consists of a catalytic and regulatory subunit that is activated by several mechanisms, including RTKs, RAS proteins, IGF-1, and cell-to-cell contact (Figure 3) [217,218]. PTEN is a lipid phosphatase tumor suppressor that negatively regulates the PI3K/AKT pathway, and the loss of function of PTEN has been shown to upregulate cancer proliferation [48,96].

The loss of PTEN function and subsequent upregulation of the PI3K-AKT pathway is frequently observed in skin cancers, including melanoma (Figure 3) [219]. 5–20% of uncultured melanomas exhibit PTEN mutations, and 28.5% of metastatic melanomas show sequence alterations in PTEN [220,221]. One study found five PIK3CA mutations in melanoma patients which include p.P539R, p.E542K, p.E545A, p.E545G, and p.E545K in exon 9 [222]. Up to 70% of melanomas demonstrated reduced PTEN expression or activation of AKT kinase [216]. The small GTPase adenosine diphosphate-ribosylation factor 6 (ARF6) has been shown to control cutaneous melanoma invasion via activation of both PI3K and AKT and a relationship has been found between ARF6 hyperactivation and metastasis of cancer [216]. One pro-invasive pathway ARF6-PI3K-AKT indicates a role for ARF6 in mediating the melanoma metastatic cascade, since aberrant activation of ARF6 leads to reduced survival in human melanoma patients [216]. Inhibitors against this pathway could be used to target RAS driven melanoma, since ARF6 may have control over RAS [216]. Mutations in the gene encoding PREX2, which interacts with PTEN, have also been implicated in activating the PI3K/AKT pathway and thus increasing carcinogenesis. Mutated PREX2 causes an activation in the guanine nucleotide exchange factor activity for RAC1, a member of the Rho family of GTPases and a PTEN binding protein. RAC1 sets off increased PI3K/AKT signaling in NRAS mutant melanoma, resulting in increased cell proliferation [223].

One clinical issue with utilizing PI3K inhibitors is their narrow therapeutic index as the pathway also modulates signals in non-cancerous cells. A solution to this dilemma lies in the development of isoform specific PI3K inhibitors to selectively target malignant cell lines [224]. LY294002 is a potent signal transduction inhibitor of the PI3K-AKT cascade. In vitro and in vivo studies have shown the ability of LY294002 to block nearly all of PI3K-AKT activity in uveal melanoma cells [36]. One study using uveal melanoma cells showed near complete inhibition of cell proliferation in cells treated with LY294002, as well as increased efficacy of LY294002 when combined with rapamycin [36]. Further studies have found that a combined dose of PIK-75 and vemurafenib, a BRAF inhibitor, halts both the PI3K/AKT and mitogen-activated protein kinase pathways, which proved to be effective against early passage cell lines derived from patient tumor samples and on melanoma cell lines resistant to either vemurafenib or dabrafenib [225]. These experiments confirm the expected effects on key signaling pathways, demonstrating the crosstalk between PI3K/AKT and MAPK pathways [225].

PI3K inhibitors may not be the best sole therapeutic agents in the treatment of melanoma. However, they may serve as an adjunctive agent to improve sensitivities to previously resistant tumors. One study showed increased sensitivities to the MEK inhibitor selumetinib and BRAF inhibitor vemurafenib in BRAF-mutant melanoma with low-pAKT expression [226]. The same study showed that PI3K inhibitors ZSTK474 or Dactolisib (BEZ235) in combination with selumetinib or vemurafenib enhanced anti-proliferative activity in BRAF-mutant cell lines [226]. The apoptotic efficacy of PI3K inhibitor Alpelisib (BYL719) has also been shown to increase under BRAF-inhibited conditions, suggesting that prolonged BRAF inhibition may lead to increased efficacy of PI3K inhibitors in treating melanoma [227]. Other studies have shown that simultaneous inhibition of the MAPK, PI3Kβ/IGF1R, and PI3Kα pathways induced apoptosis and inhibited tumor growth in BRAF-mutant and loss-of-function PTEN melanoma models [228]. Future research in targeting the MAPK and PI3K pathways may improve treatment efficacy, as both pathways can interact during signal transduction.

## 9. Conclusions

In this comprehensive review, we have described several potential pathways for molecularly targeted treatment of melanoma. **Novel** therapeutic approaches targeting genetic mutations in BRAF, MEK, RAS family of proteins, c-KIT, c-Met, VEGFR, and PI3K have been illustrated and implicated in the reduction of cell proliferation and cell survival in melanoma as well other forms of cancer. However, the issue of acquired resistance to small molecule inhibitors compromises the long-term efficacy of these strategies. Hence, a number of studies have been elucidated in this review, which seek to overcome the effects of acquired inhibitor resistance. The most common approach is the use of combination therapy to target several pathways implicated in tumorigenesis. The coupling of inhibitors against BRAF/MEK, BRAF/JNK, MEK/Plk1, MEK/pan-RAF, and PI3K/MAPK have been explored as promising approaches; however, these strategies only uncover a fraction of the therapeutic potential contained in combinatory treatment of melanoma. Additionally, immunotherapy in combination with targeted therapy has also been found to be effective. Several areas of potential future study related to important melanoma biomarkers have been highlighted throughout the review. Continued research is currently necessary for sustained innovation in the field of molecularly targeted therapy for melanoma.

## Figures and Tables

**Table 1:** Current molecularly targeted therapies, their associated targets, and their clinical status. Targets include B-Raf serine/threonine kinase (BRAF), mitogen-activated protein kinase (MEK), microphthalmia-associated transcription factor (MITF), receptor-tyrosine kinase MET (c-Met), receptor-tyrosine kinase Kit (c-KIT), vascular endothelial growth factor receptor (VEGFR), and phosphoinositide-3-kinase AKT signaling pathway (PI3K-AKT).

Inhibitor	Target	FDA Approved/Preclinical Studies and Clinical Trials	References
Vemurafenib	BRAF	FDA approved	[7,8]
Dabrafenib	BRAF	FDA approved	[9,10,11]
Trametinib	MEK	FDA approved	[9,11]
PLX8394	BRAF V600E	Preclinical studies	[12]
PLX7904	BRAF V600E	Preclinical studies	[12]
CH5552074	MITF	Preclinical studies	[10]
CH6868398	MITF	Preclinical studies	[10]
Glucocorticoids	BRAF V600E	Preclinical studies	[13]
Cobimetinib	MEK	FDA approved	[6,14]
Encorafenib	BRAF	FDA approved	[14,15]
Binimetinib	MEK	FDA approved	[14,15]
Selumetinib	MEK	Clinical Trial	[16]
CH5126766/RO5126766	MEK-RAF	Preclinical studies	[17,18]
Pimasertib	MEK	Clinical Trial	[19,20]
Imatinib	c-KIT	Clinical Trial	[21,22]
Masitinib	c-KIT	Preclinical studies	[21,23]
Sunitinib	c-KIT	Clinical Trial	[21,24]
Dasatinib	c-KIT	Clinical Trial	[21,25]
Nilotinib	c-KIT	Clinical Trial	[21,26]
Sorafenib	VEGFR, BRAF and c-KIT	Clinical Trial	[27]
Axitinib	VEGFR	Clinical Trial	[28,29]
DW10075	VEGFR	Preclinical studies	[30]
Tivantinib	c-Met	Clinical Trial	[31,32]
Cabozantinib	c-Met	Clinical Trial	[33]
Crizotinib	c-Met	Preclinical studies	[34]
Savolitinib	c-Met	Preclinical studies	[35]
LY294002	PI3K-AKT	Preclinical studies	[36,37]
Dactolisib	PI3K-AKT	Preclinical studies	[38]
Alpelisib	PI3K	Preclinical studies	[39]
